# Supplementary material for: Dynamics of Myosin II Filaments during Wound Repair in Dividing Cells
Source: Cells. 2021 May 17;10(5):1229. doi: 10.3390/cells10051229 (PMC8156316; doi:10.3390/cells10051229)
Supplement: Supplementary file 1 [file cells-10-01229-s001.zip › Supplementary files/Supplementary Table S1 final.docx]

**Dynamics of Myosin II Filaments during Wound Repair in Dividing Cells**

**Md. Istiaq Obaidi Tanvir, Go Itoh, Hiroyuki Adachi, and Shigehiko Yumura**

**Supplementary Table S1: Lists of GFP-constructs and mutants used in the present study**

Lists of GFP-constructs used in the present study

| Construct name | Source | Reference |
| --- | --- | --- |
| GFP-myosin II heavy chain | Uyeda laboratory, NBRP Nenkin | (Yumura and Uyeda, 1997a) |
| GFP-3Ala myosin II | Uyeda laboratory, NBRP Nenkin | (Yumura, 2001) |
| GFP-E476K myosin II | Uyeda laboratory, NBRP Nenkin | (Yumura and Uyeda, 1997b) |
| GFP-lifeact | Uyeda laboratory, NBRP Nenkin | (Talukder et al., 2020) |
| GFP-MHCKC | Egelhoff laboratory | (Yumura et al., 2005) |
| GFP-pakA | Firtel laboratory | (Chung and Firtel, 1999) |
| GFP-dlpA | Yumura laboratory | (Fujimoto et al., 2019) |
| GFP-clathrin light chain | O’Halloran laboratory | (Damer and O'Halloran, 2000) (Fujimoto et al., 2019) |
| GFP-PTEN (G129E) | Iijima laboratory | (Pramanik et al., 2009) |
| annexin C1-GFP | Yumura laboratory | (Talukder et al., 2020) (Pervin et al., 2018) |
| GFP-calmodulin | Yumura laboratory | (Talukder et al., 2020) |
| Dd-GCaMP6s | Ueda laboratory, NBRP Nenkin | (Tanaka et al., 2019) |
| GFP-cortexillin II | This study |  |
| GFP-GAPA | NBRP Nenkin | (Adachi et al., 1997) |

Lists of mutants used in the present study

| Mutant name | Source | Reference |
| --- | --- | --- |
| AX2 (wild type) | Yumura laboratory |  |
| myosin II heavy chain null (HS1) | DictyBase Stock Center | (Manstein et al., 1989) |
| MHCKC null | Egelhoff laboratory | (Yumura et al., 2005) |
| PAKA null | Firtel laboratory | (Chung and Firtel, 1999) |
| DlpA null | Yumura laboratory | (Fujimoto et al., 2019) |
| clathrin heavy chain null | O’Halloran laboratory | (Ruscetti et al., 1994) |
| cortexillin II null | DictyBase Stock Center | (Faix et al., 2001) (Weber et al., 1999) |
| PTEN null | Iijima laboratory | (Iijima and Devreotes, 2002) (Janetopoulos et al., 2005) |
| GapA null | Adachi laboratory | (Adachi et al., 1997) |

**References**

**Adachi, H., Takahashi, Y., Hasebe, T., Shirouzu, M., Yokoyama, S. and Sutoh, K.** (1997). Dictyostelium IQGAP-related protein specifically involved in the completion of cytokinesis. *J Cell Biol.* **137,** 891-898.

**Chung, C. Y. and Firtel, R. A.** (1999). PAKa, a putative PAK family member, is required for cytokinesis and the regulation of the cytoskeleton in Dictyostelium discoideum cells during chemotaxis. *J Cell Biol.* **147,** 559-576.

**Damer, C. K. and O’Halloran, T. J.** (2000). Spatially regulated recruitment of clathrin to the plasma membrane during capping and cell translocation. *Mol Biol Cell.* **11,** 2151-2159.

**Faix, J., Weber, I., Mintert, U., Köhler, J., Lottspeich, F. and Marriott, G.** (2001). Recruitment of cortexillin into the cleavage furrow is controlled by Rac1 and IQGAP-related proteins. *EMBO J.* **20,** 3705-3715.

**Fujimoto, K., Tanaka, M., Rana, A. Y. K. M. M., Jahan, M. G. S., Itoh, G., Tsujioka, M., Uyeda, T. Q. P., Miyagishima, S. Y. and Yumura, S.** (2019). Dynamin-Like Protein B of Dictyostelium Contributes to Cytokinesis Cooperatively with Other Dynamins. *Cells.* **8,** 781.

**Iijima, M. and Devreotes, P.** (2002). Tumor suppressor PTEN mediates sensing of chemoattractant gradients. *Cell.* **109,** 599-610.

**Janetopoulos, C., Borleis, J., Vazquez, F., Iijima, M. and Devreotes, P.** (2005). Temporal and spatial regulation of phosphoinositide signaling mediates cytokinesis. *Dev Cell.* **8,** 467-477.

**Manstein, D. J., Titus, M. A., De Lozanne, A. and Spudich, J. A.** (1989). Gene replacement in Dictyostelium: generation of myosin null mutants. *EMBO J.* **8,** 923-932.

**Pervin, M. S., Itoh, G., Talukder, M. S. U., Fujimoto, K., Morimoto, Y. V., Tanaka, M., Ueda, M. and Yumura, S.** (2018). A study of wound repair in Dictyostelium cells by using novel laserporation. *Sci Rep.* **8,** 7969.

**Pramanik, M. K., Iijima, M., Iwadate, Y. and Yumura, S.** (2009). PTEN is a mechanosensing signal transducer for myosin II localization in Dictyostelium cells. *Genes Cells.* **14,** 821-834.

**Ruscetti, T., Cardelli, J. A., Niswonger, M. L. and O’Halloran, T. J.** (1994). Clathrin heavy chain functions in sorting and secretion of lysosomal enzymes in Dictyostelium discoideum. *J Cell Biol.* **126,** 343-352.

**Talukder, M. S. U., Pervin, M. S., Tanvir, M. I. O., Fujimoto, K., Tanaka, M., Itoh, G. and Yumura, S.** (2020). Ca^2+^-Calmodulin Dependent Wound Repair in Dictyostelium Cell Membrane. *Cells.* **9,** 1058.

**Tanaka, Y., Jahan, M. G. S., Kondo, T., Nakano, M. and Yumura, S.** (2019). Cytokinesis D is Mediated by Cortical Flow of Dividing Cells Instead of Chemotaxis. *Cells.* **8,** 473.

**Weber, I., Gerisch, G., Heizer, C., Murphy, J., Badelt, K., Stock, A., Schwartz, J. M. and Faix, J.** (1999). Cytokinesis mediated through the recruitment of cortexillins into the cleavage furrow. *EMBO J.* **18,** 586-594.

**Yumura, S.** (2001). Myosin II dynamics and cortical flow during contractile ring formation in *Dictyostelium* cells. *J Cell Biol.* **154,** 137-146.

**Yumura, S. and Uyeda, T. Q.** (1997). Myosin II can be localized to the cleavage furrow and to the posterior region of Dictyostelium amoebae without control by phosphorylation of myosin heavy and light chains. *Cell Motil Cytoskeleton.* **36,** 313-322.

**Yumura, S. and Uyeda, T. Q.** (1997). Transport of myosin II to the equatorial region without its own motor activity in mitotic Dictyostelium cells. *Mol Biol Cell.* **8,** 2089-2099.

**Yumura, S., Yoshida, M., Betapudi, V., Licate, L. S., Iwadate, Y., Nagasaki, A., Uyeda, T. Q. and Egelhoff, T. T.** (2005). Multiple myosin II heavy chain kinases: roles in filament assembly control and proper cytokinesis in Dictyostelium. *Mol Biol Cell.* **16,** 4256-4266.
