# Supplementary material for: Dynamics of Myosin II Filaments during Wound Repair in Dividing Cells
Source: Cells. 2021 May 17;10(5):1229. doi: 10.3390/cells10051229 (PMC8156316; doi:10.3390/cells10051229)
Supplement: Supplementary file 1 [file cells-10-01229-s001.zip › Supplementary files/Supplementary Fig.S1.docx]

**Dynamics of Myosin II Filaments during Wound Repair in Dividing Cells**

**Md. Istiaq Obaidi Tanvir, Go Itoh, Hiroyuki Adachi, and Shigehiko Yumura**

**Supplementary Figure S1**


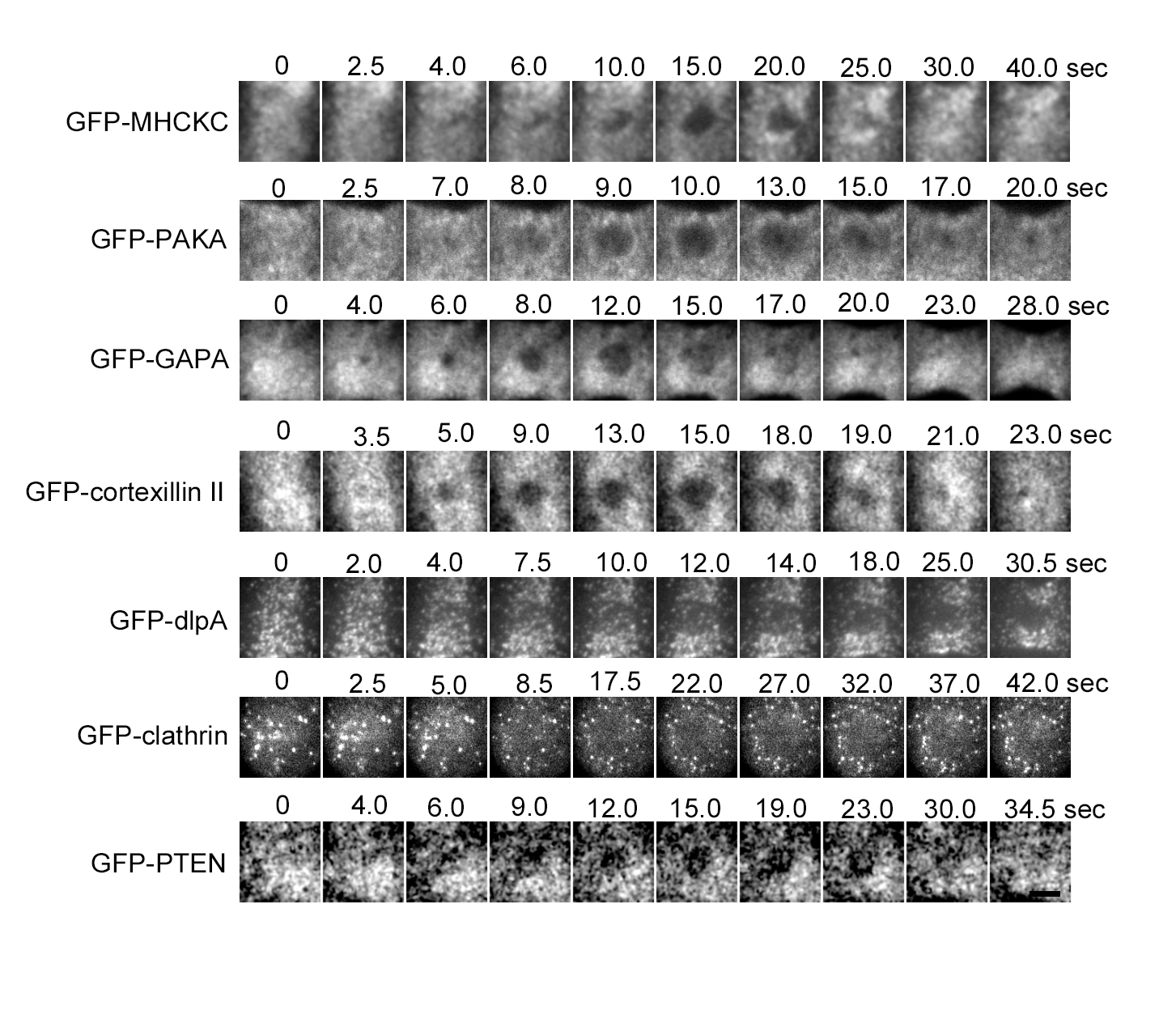


**Figure S1. Dynamics of several furrow-localizing proteins at wound sites**

Typical sequences of fluorescence images of GFP-MHCKC, GFP-PakA, GFP-GAPA, GFP-cortexillin II, GFP-dlpA, GFP-clathrin, and GFP-PTEN in the cleavage furrow upon wounding. Images show only wound sites. Clathrin is not furrow-localizing protein. Bars, 2 μm.
